# Rescue of naïve porcine circovirus type 3 and its pathogenesis in CD pigs

**DOI:** 10.1128/jvi.00341-25

**Published:** 2025-05-12

**Authors:** Baoge Zhang, Jinshuang Cai, Chenguang Zhu, Yuyu Zhang, Jiaqiang Wu, Yufeng Li

**Affiliations:** 1 Key Laboratory of Bacteriology, Ministry of Agriculture, College of Veterinary Medicine, Nanjing Agricultural University 261674, Nanjing, China; 2 Shandong Key Laboratory of Animal Disease Control and Breeding, Institute of Animal Science and Veterinary Medicine, Shandong Academy of Agricultural Sciences 722363 , Jinan, China; Cornell University Baker Institute for Animal Health, Ithaca, New York, USA

**Keywords:** PCV3, TEM, PBMCs, scRNA-seq, infection traits

## Abstract

The pathogenesis of naïve porcine circovirus type 3 (PCV3) in pigs is not accurately elucidated due to virus isolation failure *in vitro*. In this study, naïve PCV3 was successfully rescued and used to infect cesarean-derived (CD) pigs. The PCV3 genome was synthesized, cloned, and inserted into the pBluescript SK vector. PK-15 cells transfected with the recombinant plasmid pSK-PCV3 were passaged, and Cap protein expression was confirmed by immunofluorescence assay (IFA) and western blotting. Virus particles (~20 nm) were observed via transmission electron microscopy (TEM). The viral growth curve was plotted to determine the replication of the virus within the cells. Nocodazole treatment demonstrated that PCV3 replication is dependent on microtubule polymerization in the cell. Cells infected with PCV3 or PCV3-positive clinical samples (wPCV3) showed only cytoplasmic fluorescence. PCV3 can successfully infect CD pigs, resulting in persistent viremia, and increased viral loads in tissues and an antibody delay were observed. Inflammatory lesions were also observed in the lungs, lymph nodes, livers, and intestines of infected pigs. Stimulation of peripheral blood mononuclear cells (PBMCs) with virus-like particles (VLPs) containing the Cap protein significantly inhibited cell proliferation. scRNA-seq revealed a significant reduction in T helper 2 (Th2) cells and the migration of T helper 1 (Th1) cells toward the late differentiation stage following infection. In particular, the decrease in Th2 cells may indicate impaired humoral immunity, leading to delayed antibody production and immunosuppression. Our study is the first to observe PCV3 via TEM and to elucidate its immunosuppressive mechanisms in CD pigs.

**IMPORTANCE:**

This study is of great significance as it successfully rescued naïve PCV3 and, for the first time, observed clear PCV3 viral particles using transmission electron microscopy. The successful infection of CD pigs deepened our understanding of its pathogenic mechanisms. The results revealed key aspects of viral replication, the impact of the virus on immune responses, and associated inflammatory lesions in various tissues. Notably, the study found that the reduction of Th2 cells leads to impaired humoral immunity and delayed antibody production, which may provide valuable insights for vaccine development and swine disease management.

## INTRODUCTION

Porcine circovirus (PCV) is a circular, single-stranded DNA virus that belongs to the Circoviridae family and the Circovirus genus, including PCV1–4 (1). To date, the pathogenesis of PCV2 has been widely studied, but few studies have investigated the pathogenesis of PCV3. The genome of PCV3 is approximately 2,000 nt in length and contains three major open reading frames (ORFs): ORF1, ORF2, and ORF3 (2, 3). In 2016, Palinski et al. first discovered PCV3 through metagenomic analysis in diseased sows with porcine dermatitis and nephropathy syndrome (PNDS) and aborted fetuses (4). Since then, viral detection of PCV3 has been reported in many countries across Europe, Asia, and the Americas, indicating the rapid global spread of the virus (5–12). In addition to being detected in pigs, PCV3 has also been identified in canids and other mammalian species (13, 14). Cui et al. (15) proposed that PCV3 originated from bats and that birds may serve as important intermediate hosts that transmit bat circoviruses to pigs, thereby increasing the risk of cross-species transmission and affecting public health security. PCV3 can be vertically transmitted, crossing the placental barrier and replicating within fetal tissues, leading to high viral loads and potentially causing stillbirths and mummified fetuses in pregnant sows (16–22). The presence of persistent PCV3 viremia increases the transmission of this virus (21, 23–25). PCV3 can be detected in pigs at different stages, with the highest viral loads found in mummified fetuses, suggesting that the virus may contribute to reproductive failure (26, 27). PCV3 infection may cause immunosuppression in pigs, leading to diminished immune responses (28).

Krüger et al. (29) investigated the transmission of PCV3 through xenotransplantation, demonstrating that PCV3-positive donor pigs could transmit the virus to baboons following heart transplantation. Their study provided the first evidence of PCV3 cross-species transmission, as viral DNA was detected in multiple organs of transplanted baboons. Notably, higher viral loads correlated with longer survival times, suggesting PCV3 persistence and replication in the host. While PCV3 was transmissible to baboons, its ability to infect humans remains uncertain, highlighting the need for further research into PCV3 replication, immune response, and zoonotic potential. Although PCV3 is transmissible in baboons, its ability to infect humans remains unclear, necessitating further in-depth studies on PCV3. Currently, isolating and purifying PCV3 from clinical samples poses significant challenges; therefore, rescuing PCV3 using an infectious clone represents a valuable alternative approach.

scRNA-seq, a powerful sequencing tool, achieves high-throughput and multidimensional analysis of individual cells by describing cell subpopulation classification and cellular heterogeneity. It enables a more precise exploration of the functions of different cell types and provides deeper insights into both scientific research and clinical applications (30). Herrera-Uribe et al. (31) utilized signature gene sets from human Hemopedia data to assess the relative enrichment of expressed genes in pig cells. They integrated pig scRNA-seq data with a public human scRNA-seq data set, further validating the similarities between human and pig data. Single-cell sequencing technology is widely applied in the research of various significant pathogens, including human viruses such as the influenza virus and the COVID-19 virus, as well as porcine viruses such as the porcine epidemic diarrhea virus, the African swine fever virus, and the porcine reproductive and respiratory syndrome virus (32–35). In single-cell sequencing, clustering PBMCs into distinct groups is important for understanding the correlation of gene expression among cells and the functional characteristics of the immune system. Through further investigation of these clusters, we can gain further insight into the functional and differentiation relationships of different cell types, thereby aiding in the elucidation of underlying biological mechanisms.

Infectious clones serve as indispensable tools for studying the molecular biology of porcine circoviruses (PCVs), enabling precise investigations into viral replication, pathogenesis, and host-virus interactions (36). The replication of PCVs is driven by a rolling-circle replication (RCR) mechanism, initiated by the Rep protein, which facilitates viral genome amplification within the nucleus of infected cells (37). The Cap protein plays a crucial role in virion assembly and host immune recognition (38). Compared to PCV1 and PCV2, PCV3 exhibits significant genetic divergence, which may contribute to differences in host tropism, virulence, and immune evasion strategies. The successful rescue of naïve PCV3 will provide indispensable biological materials for conducting pig infection experiments to reveal the pathological mechanisms of this virus, which will be helpful for subsequent vaccine development and xenotransplantation, overcoming the bottleneck in pig infection experiments and human circovirus studies.

## MATERIALS AND METHODS

### Construction of the recombinant plasmid

The full-length PCV3 genome (GenBank: PP196470.1) was inserted into the pBluescript II SK+ (pSK) vector using the EcoRI and SpeI restriction sites, with the insertion direction matching the replication origin (Ori) of the vector. The recombinant plasmid was designated pSK-PCV3 and identified by gene sequencing and BLAST in the NCBI database.

### Rescue of naïve PCV3

PK-15 cells were transfected with pSK-PCV3 and passaged continuously. The transfection process was performed as follows: PK-15 cells were seeded in a six-well plate until they reached approximately 80% confluence. After the supernatant was discarded, the cell monolayer was washed three times with sterile PBS, and 1 mL of DMEM (Gibco) free from penicillin/streptomycin and serum was added and incubated in 5% CO_2_ at 37°C. 4 µL of Lip2000 transfection reagent (Thermo Scientific) and 4 µg of the pSK-PCV3 recombinant plasmid were separately mixed with 50 µL of DMEM and incubated for 5 min. The two mixtures were then mixed and incubated for an additional 20 min. The mixture was added to the prepared PK-15 cell monolayer. After 4 h, 1 mL of complete growth medium was added. The cells were further incubated for 24–48 h before proceeding with cell passage.

### Indirect IFA assay

For the detection of transfected cells, virus-carrying cells were continuously passaged three times and then seeded into 96-well plates. After 12 h of incubation, the cells were fixed for immunofluorescence detection. PK-15 cells transfected with the empty vector were used as the negative control. For the detection of infected cells, uninfected PK-15 cells served as the negative control, while PK-15 cells infected with wPCV3 were used as the positive control. PK-15 cells infected with rescued PCV3 were designated as the experimental group. PK-15 cells were infected separately with PCV3 and wPCV3 at a viral copy number of 1 × 10^8^, and detection was performed at 24, 36, and 48 hours post-infection.

After the supernatant was discarded, the cells were fixed with 4% paraformaldehyde for 20 min. Subsequently, the cells were washed three times with PBST (phosphate-buffered saline with Tween 20) for 5 min each. The cells were then treated with 0.5% Triton-X-100 at room temperature for 20 min, followed by three washes with PBST as described above. The monoclonal antibody 2E6 (data published in Chinese) prepared in the laboratory was added and incubated overnight at 4°C. After three washes with PBST, the cells were incubated with an FITC-conjugated mouse secondary antibody (Cell Signaling Technology) at 37°C for 1 h. The secondary antibody solution was then discarded, and each well was stained with 50 µL of DAPI. After a 5 min incubation at room temperature, the cells were washed three times with PBST. Finally, fluorescence microscopy was used to observe the cells.

### Western blot analysis

PK-15 cells transfected with the eukaryotic plasmid pCAGGS-PCV3-Cap (stored in our laboratory) were collected as a positive control. The monoclonal antibody 2E6 was used as the primary antibody, and the HRP-labeled goat anti-mouse antibody was used as the secondary antibody to detect the expression of PCV3-Cap protein in the third passage of PK-15 cells carrying the virus.

### Genome copy number determination

A recombinant plasmid was constructed using the ORF2 gene encoding the PCV3-Cap protein. The primer sequences for amplification were as follows: forward, TTAGAGAACGGACTTGTAACGAATC; reverse, ATGAGACACAGAGCTATATTC.

The full-length PCV3-ORF2 gene was ligated into a T-vector and confirmed by sequencing to construct a recombinant plasmid. The plasmid concentration was determined using a K5500 microvolume spectrophotometer, and the plasmid copy number was calculated as 4.8 × 10^11^ copies/μL. Standard plasmids with concentrations ranging from 4.8 × 10^9^ to 4.8 × 10^1^ copies/μL were used as templates for real-time fluorescence quantitative PCR amplification with a melting temperature (Tm) of 57°C to establish a standard curve. The number of viral genome copies (gc) was determined based on the established standard curve.

### Viral growth kinetic curve

To determine the viral growth kinetics, viral copy numbers were measured in virus-carrying passaged cells at 12, 24, 36, 48, 60, and 72 h. The collected samples were treated with DNase at 37°C for 30 min to effectively reduce potential contamination from the original plasmid. RNA was extracted from the cells and reverse transcribed into cDNA. Viral copy numbers were quantified using RT-qPCR to accurately measure the number of viral genomes in the infected cells. The data were then plotted as a viral growth curve to characterize the replication dynamics of the virus over time.

### Proliferation of PCV3

Nocodazole (Absin Biotechnology) was prepared as a 1 mM stock solution using DMSO. The final working concentrations required were 10 µM, 20 µM, and 30 µM. To exclude the impact of changes in cell viability on PCV3 proliferation after nocodazole treatment, cell viability following drug treatment was assessed using a CCK-8 assay. The experimental groups included a control group, a DMSO group, and a nocodazole treatment group. After cell transfection, when the confluence reached 70%, the cells were treated with different working concentrations of nocodazole (10 µM, 20 µM, or 30 µM) for 4 h. After the medium was changed, the cells were further cultured for 24 h, after which the cells were collected for DNA extraction to determine the viral copy number. Protein lysates were prepared, and western blot analysis was performed to detect Cap protein expression.

### Comparison of infection characteristics between PCV3 and wPCV3

wPCV3 refers to the PCV3-positive sample previously isolated, identified, and preserved by our laboratory, where only PCV3 tested positive, and other pathogens tested negative. The sample was homogenized, and the viral copy number was determined. PK-15 cells were separately infected with PCV3 with a viral copy number of 1 × 10^8^ and with wPCV3. At different time points after infection, an IFA was performed to observe the infected cells, and viral copy numbers were calculated.

### CD pig infection experiment

Eight 28-day-old PCV-negative CD piglets were randomly and evenly divided into an infection group and a control group. The inoculation was administered via intranasal injection and intramuscular injection into the right neck triangle area, with a dosage of 5 × 10^11^ copies per piglet in the infection group, 2 mL in each nostril, and 1 mL by intramuscular injection, while the control group received an equivalent volume of sterile DMEM instead. The viral inoculum used in the infection group was from a successfully rescued PCV3.

### Antibody detection

We established a blocking ELISA method to detect PCV3 using PCV3-Cap expressed in a prokaryotic system as the coating antigen and 2E6 monoclonal antibody as the detection antibody. When the percentage inhibition (PI) was ≥28.30%, the result was considered positive. Serum was tested at 7, 14, 21, 28, and 35 d after infection.

### Immunohistochemical staining

The immunohistochemical staining process involves several key steps to ensure precise detection of the target antigen. Tissues were first fixed in formalin to preserve their structure and then sliced into thin sections. After deparaffinization with xylene and rehydration through a graded ethanol series (100%, 95%, 85%, and 70%), sections were treated with antigen retrieval solution (citrate buffer, pH 6.0) and heated at 95°C for 20 min to unmask the antigenic sites. Non-specific binding was blocked by incubating the tissue sections with 5% bovine serum albumin (BSA) for 30 min at room temperature. The 2E6 monoclonal antibody was used as the primary antibody at a 1:1,000 dilution. Sections were incubated with the primary antibody overnight at 4°C. After washing with PBS, the tissue sections were incubated with biotinylated secondary antibody (goat anti-mouse IgG) at a 1:500 dilution for 30 min at 37°C. The antigen-antibody complex was visualized using DAB as the chromogenic substrate. To counterstain the sections, hematoxylin was applied, followed by dehydration in increasing concentrations of ethanol (75%, 85%, 95%, and 100%) and clearing in xylene. Finally, the sections were mounted using a water-soluble mounting medium (neutral gum) and observed under a microscope.

PCV3 virus is localized in the cytoplasm and nucleus of infected cells. After DAB staining, brown signals were observed in the cytoplasm and nucleus of the infection group, in stark contrast to the control group.

### PBMC proliferation assay

At 14 and 28 days post-infection, 5–10 mL of aseptic blood samples were collected from the experimental pigs via the anterior vena cava and anticoagulated with EDTA. PBMCs were isolated using pig PBMC isolation medium through density gradient centrifugation. The isolated PBMCs were then plated at a density of 2 × 10⁵ cells per well onto a 96-well cell culture plate. After incubation at 37°C in a 5% CO₂ incubator for 4 h, the PBMCs were stimulated with VLPs derived from the Cap protein expressed in insect cells (stored in our laboratory) at a final concentration of 5 µg/mL. Concanavalin A (ConA) was used as a positive control, at a final concentration of 5 µg/mL. The 96-well plate was then incubated at 37°C in a 5% CO₂ incubator for 72 h. After incubation, CCK-8 reagent was added, and the OD_450_ values were measured to calculate the stimulation index (SI).

### Sample preparation for 10× scRNAseq

On the 14th d post-infection with PCV3, blood samples from the control and infected groups were collected using EDTA anticoagulant tubes. PBMCs were isolated using a porcine PBMC isolation kit (Solarbio), which achieved a cell viability of approximately 90%. Subsequently, the cell density was adjusted to 1 × 10^6^ cells/mL. High-quality single-cell suspensions were generated using a 10× Genomics v.3 kit (10× Genomics, USA). Single-cell encapsulation, complementary DNA (cDNA) library synthesis, RNA sequencing, and data analysis were completed by Tiangen (Beijing, China).

### 10× scRNA-seq data analysis

The raw scRNA-seq data were aligned, filtered, and normalized using Cell Ranger software (10× Genomics). Reads mapped to the transcriptome were used for unique molecular identifier (UMI) counting. The results obtained from the Cell Ranger analysis included read and UMI information and expression data extracted using Seurat software for single-cell expression clustering and analysis of gene expression levels. In addition, data from multiple samples can be integrated for comparison. Seurat’s t-distributed stochastic neighbor embedding (t-SNE) or UMAP is used for the visualization and exploration of these data sets. Other data analyses, including standardization, differential gene expression, and marker gene selection, were also implemented through Seurat.

### Statistical analysis

The data were analyzed using GraphPad Prism 8.0 software. The results are presented as the means ± SDs. Paired t tests and one-way ANOVA were used for statistical comparisons. The data were considered significant at *P < 0.05, **P < 0.01, and ***P < 0.001.

## RESULTS

### Identification and growth kinetics of PCV3

**Fig 1:**
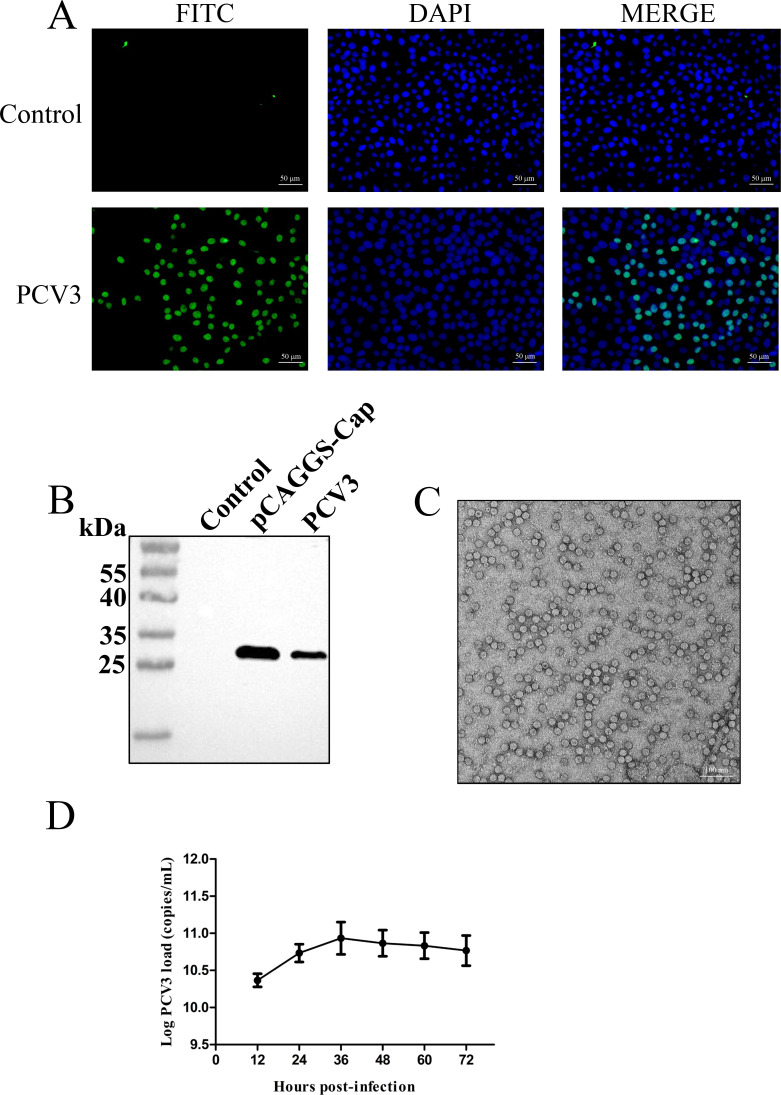
PCV3 identification using IFA, Western blot, transmission electron microscopy, and viral growth kinetic curve. (**A**) IFA detection (400×) of PCV3. Immunostaining of transfected cells passaged to the third generation with virulence after transfection using monoclonal antibody 2E6 to visualize PCV3-specific nuclear fluorescence (green) and DAPI-stained nuclei (blue). (**B**) Western blot analysis of PCV3. PK-15 cells were used as a negative control, while PK-15 cells transfected with the eukaryotic plasmid pCAGGS-PCV3-Cap were used as a positive control. The size of the target band for PCV3-Cap was consistent with that of the positive control. (**C**) Transmission electron microscopy observation (73,000×). Circular virus particles with a diameter of approximately 20 nm are visible under transmission electron microscopy. (**D**) Viral growth kinetic curve. Viral copy numbers were determined in virulent passaged cells at 12, 24, 36, 48, 60, and 72 h after passaging, with the highest viral copy number at 36 h.

After PK-15 cells were transfected with pSK-PCV3, the first passage was conducted 48 h post-transfection. At this stage, PCV3 viral particles were present in the cells, and subsequent passages were referred to as virus-carrying cell passages. The viral supernatant obtained from the first passage was designated as P1, with subsequent passages labeled accordingly. In this study, viral samples were collected after three passages (P3). The collected samples were treated with DNase at 37°C for 30 min to effectively reduce potential contamination from the original plasmid. PCV3-specific monoclonal antibodies were used for identification. The IFA results showed that specific fluorescence was observed only in the cell nucleus (Fig. 1A). Specific Cap protein was detected by western blot in comparison with that in the mock-transfected cells (Fig. 1B). Taken together, these results indicated the successful rescue of the naïve PCV3 virus by confirming the expression of the Cap protein. The continuously passaged cells harboring the virus were repeatedly frozen and thawed to release viral particles. The virus particles were pelleted by ultracentrifugation, purified by sucrose density gradient centrifugation and ion exchange, and subjected to observation by transmission electron microscopy. We found that the PCV3 viral particles were spherical and approximately 20 nm in size (Fig. 1C).

To gain a detailed understanding of the viral replication characteristics, viral copy numbers were measured in virus-carrying passaged cells at 12, 24, 36, 48, 60, and 72 h (determination method refer to Fig. S1), and a growth kinetics curve was generated (Fig. 1D). The highest viral copy number was observed at 36 h, indicating that viral replication peaked at this time point.

### Genome copy number determination of PCV3

The total DNA of PK-15 cells harboring the PCV3 virus was extracted and used as a template, followed by PCR amplification using specific primers. The results revealed that PCV3 was able to persist within cells for up to 25 generations (Fig. S1A and B).

To determine the number of genome copies of PCV3, the ORF2 gene was used for absolute quantitative PCR. The standard curve was determined to be y = −3.3342 x +41.851, with a correlation coefficient (R²) of 0.9989 (Fig. S2A and B). As low as 4.8 copies/μL were detected by real-time fluorescence quantitative PCR (Fig. S2C and D).

The copy number of PCV3 was determined starting from the third generation (Table 1). As the cells were continuously passaged, the viral copy number gradually decreased from 1.2 × 10^11^ to 1.2 × 10^3^ copies/mL, indicating successful rescue of the PCV3 virus but the inability to proliferate within PK-15 cells effectively.

**TABLE 1:** Viral copy numbers in different generations^*a*^

Times of passage	Number of copies (copies/mL)	Times of passage	Number of copies (copies/mL)
P3	1.2 × 10^11^ ± 2.02	P15	2.1 × 10^7^ ± 1.62
P4	1.8 × 10^10^ ± 1.50	P16	2.5 × 10^6^ ± 2.08
P5	1.5 × 10^9^ ± 3.18	P17	2.4 × 10^6^ ± 1.19
P6	2.3 × 10^8^ ± 4.72	P18	2.4 × 10^6^ ± 1.18
P7	2.3 × 10^8^ ± 5.60	P19	2.2 × 10^6^ ± .87
P8	2.1 × 10^8^ ± 1.10	P20	2.1 × 10^6^ ± .68
P9	2.1 × 10^8^ ± 1.56	P21	1.5 × 10^5^ ± .64
P10	2.0 × 10^8^ ± .62	P22	1.1 × 10^5^ ± 1.28
P11	1.9 × 10^8^ ± 2.75	P23	2.3 × 10^4^ ± 2.48
P12	1.2 × 10^8^ ± 1.06	P24	1.1 × 10^4^ ± .46
P13	1.1 × 10^8^ ± 3.84	P25	1.2 × 10^3^ ± .49
P14	2.3 × 10^7^ ± 1.50		

^*a*^ The data are presented as the means ± SDs, *n* = 3.

### PCV3 proliferation

**Fig 2:**
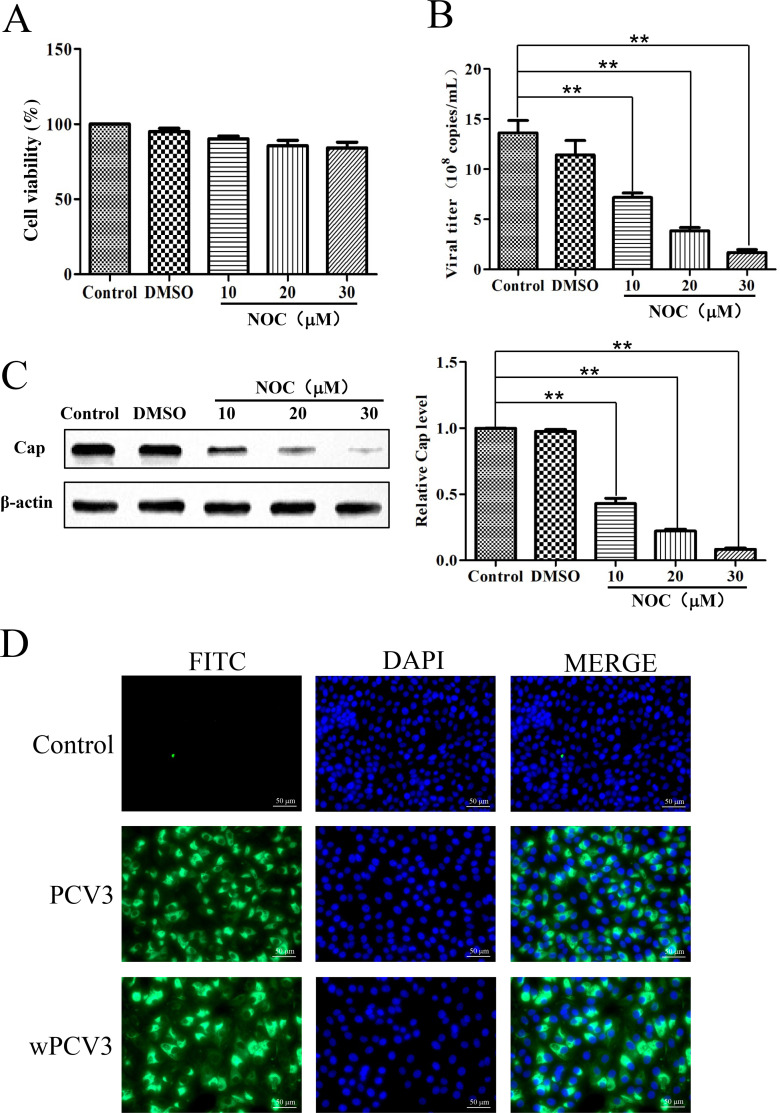
PCV3 replication is associated with microtubule polymerization, and the infection of PK-15 cells with PCV3 and wPCV3 was detected by IFAs. (**A**) Cell viability after drug treatment was determined using the CCK-8 assay, and there was no significant difference in cell viability between the drug-treated group and the mock-treated group. (**B and C**) Compared to those in the control group, the viral titer and cap expression in the drug-treated group were significantly decreased in a dose-dependent manner. The data are presented as the means  ±  SDs. *P < 0.05; **P < 0.01. (**D**) Cytoplasmic fluorescence (green) was observed in the PCV3- and wPCV3-infected PK-15 cells at 24 h post-infection using a 2E6 monoclonal antibody, while the cell nuclei were stained with DAPI (blue).

After nocodazole treatment, cell viability was determined using the CCK-8 assay. There was no significant difference between the drug-treated cells and mock-treated cells (*P*>0.05 ) (Fig. 2A). Compared with those in the control group, the viral titer and cap expression in the drug-treated group significantly decreased in a dose-dependent manner (*P* < 0.01) (Fig. 2B and C). These data suggest that PCV3 replication is dependent on microtubule polymerization in the cell.

Following the infection of PK-15 cells with PCV3 and wPCV3, cytoplasmic fluorescence was observed in both infected cell lines using IFA (Fig. 2D; Fig. S3 and 4). Quantification of viral genome copy numbers at different time points post-infection revealed a decreasing trend with increasing infection time (Table 2). These findings collectively indicate that the two viruses have similar infection characteristics and that both are unable to proliferate within PK-15 cells.

**TABLE 2:** Viral copy numbers at different times post-infection^*a*^

Hours post-infection	12 h	24 h	36 h	48 h
Copies of PCV3 (copies/mL)	2.9 × 10^7^ ± .78	2.3 × 10^7^ ± .81	2.2 × 10^7^ ± .92	1.8 × 10^7^ ± 1.19
Copies of wPCV3 (copies/mL)	2.8 × 10^7^ ± 1.31	2.2 × 10^7^ ± .80	1.7 × 10^7^ ± .74	1.2 × 10^7^ ± .85

^*a*^ The data are presented as the means ± SDs, *n* = 3.

### Infection of CD pigs with PCV3

**Fig 3:**
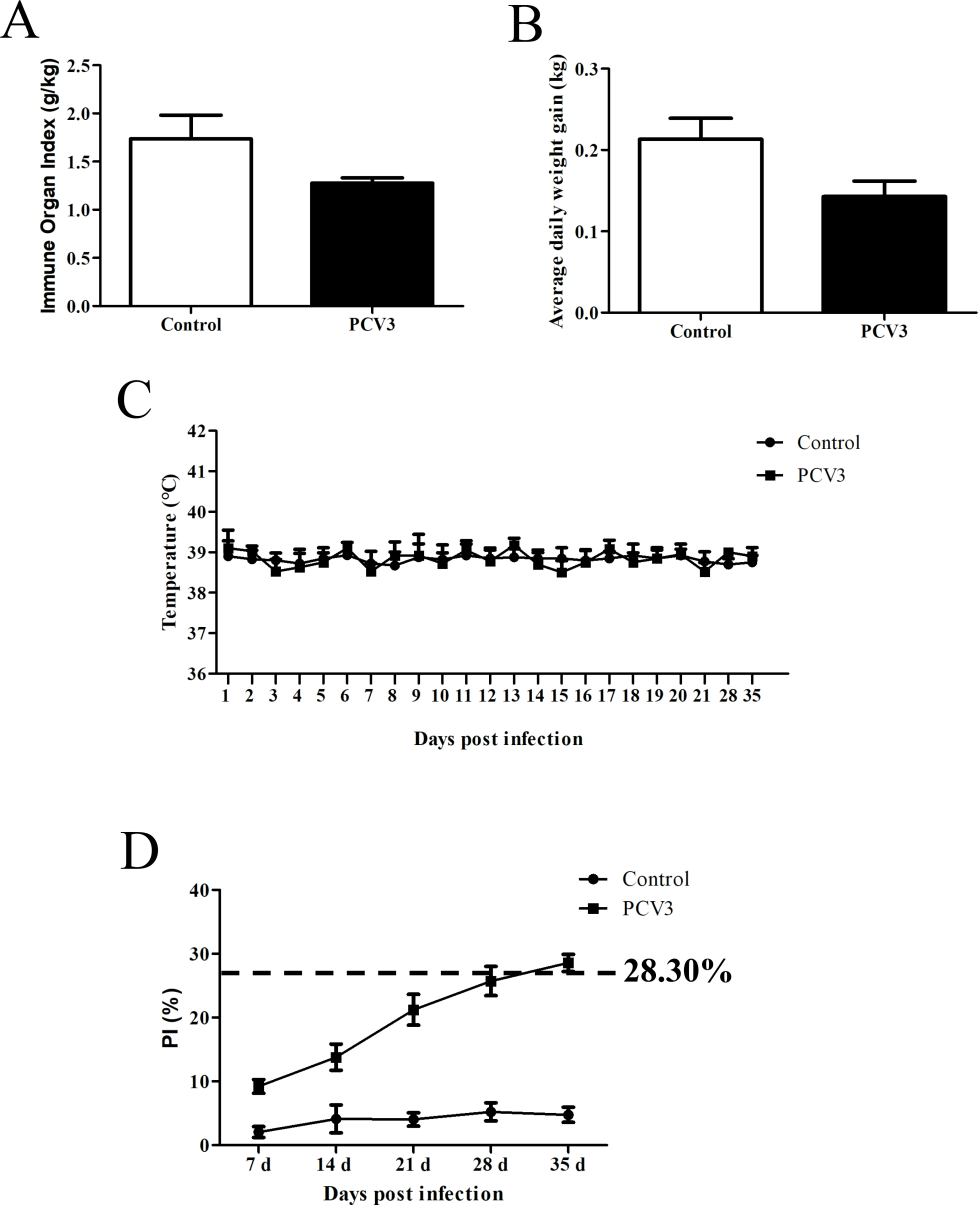
Clinical performance and immunological responses. (**A**) Spleen organ index at 35 d post-infection. (**B**) AWG within 35 d post-infection. (**C**) Temperature change curve within 35 d post-infection. (**D**) Detection of antibody levels within 35 d post-infection using the block ELISA method, expressed as percent inhibition (PI).

Compared to those in the control group, the pigs in the experimental group exhibited coarse fur, but no other significant clinical signs were observed. Thirty-five days post-PCV3 challenge, the control and experimental group pigs were euthanized to examine whether there were pathological lesions in tissues and organs. Simultaneously, various samples were collected for subsequent experimental analysis. The infected pigs exhibited mild interstitial pneumonia and slight pulmonary edema, along with slight splenic atrophy, and no significant lesions were observed in other organs. Compared to that in the control group, the spleen organ index (the ratio of spleen weight to body mass) in the infected group decreased by 0.46 g/kg (Fig. 3A). All organs remained normal in the control group. The body weights were recorded at 1 and 35 d post-infection to calculate the average daily weight gain (AWG). The data revealed that the AWG in the infected group was 70 g/d lower than that in the control group (Fig. 3B), with no significant differences in body temperature between the two groups (Fig. 3C). Antibody titers were assessed, and antibodies remained undetectable within 28 d post-infection and became weakly positive by d 35, with a blocking rate of 28.58%. By contrast, antibody levels in the control group remained negative throughout the study period (Fig. 3D).

**Fig 4:**
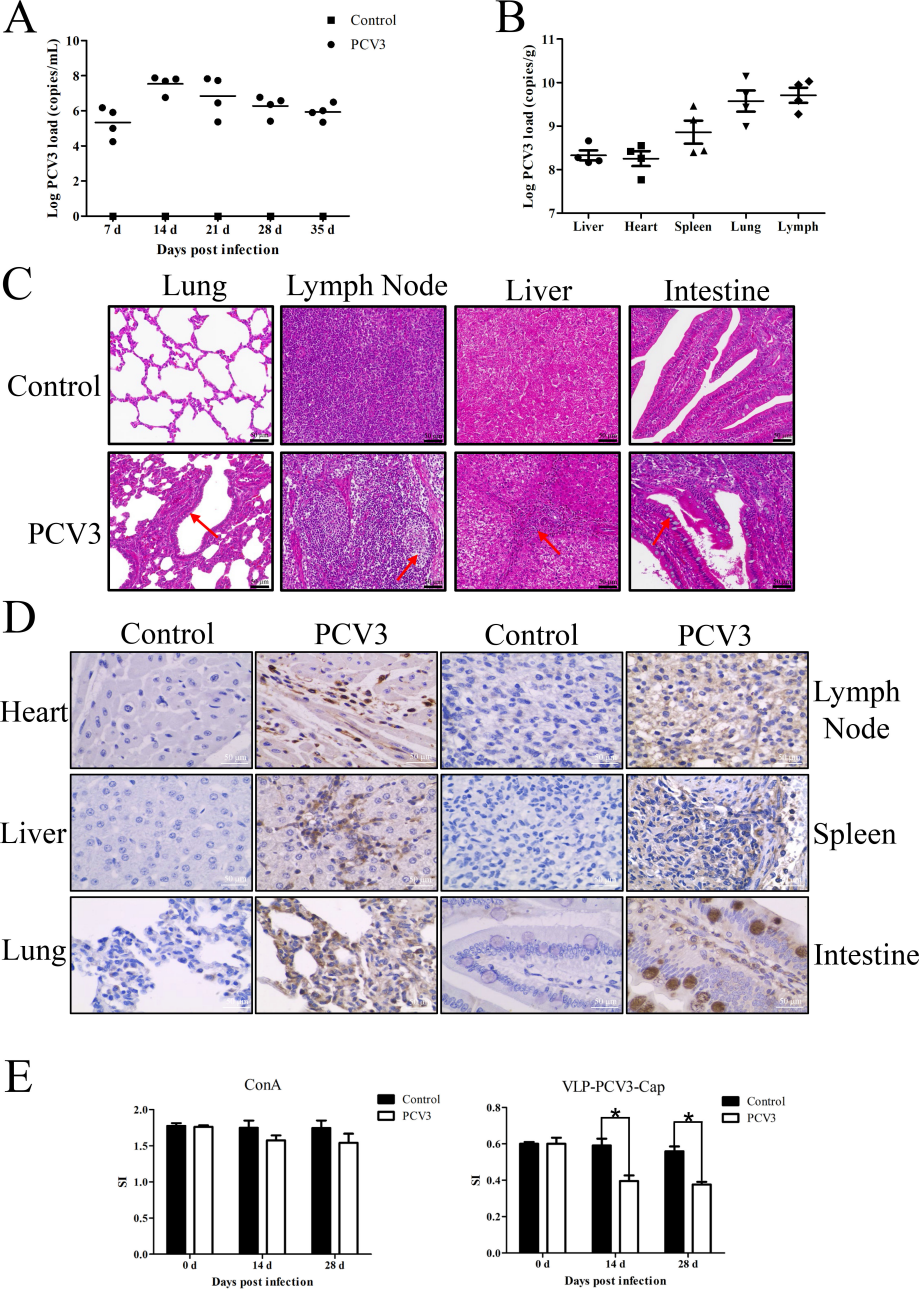
Infection characteristics of PCV3. Detection of viral viremia in sera (**A**) and viral load in tissues (**B**) using a real-time PCR method. (**C**) Histopathological lesions in the lungs, lymph nodes (LNs), liver, and intestine 35 d post-infection, with lesion sites indicated by red arrows (400×). (**D**) At 35 d post-infection, immunohistochemical analysis of the heart, liver, lungs, lymph nodes (LNs), spleen, and intestines was performed. Brown-specific punctate deposits were observed following DAB staining (400×). (**E**) At 14 and 28 days post-infection, PBMCs were separately stimulated with ConA and VLP-PCV3-Cap. After stimulation with ConA, there was no significant difference in cell proliferation between the infected group and the control group. However, following stimulation with VLP-PCV3-Cap, there was a significant difference between the two groups, and cell proliferation was inhibited. The data are presented as the means  ±  SDs. *P < 0.05; **P < 0.01.

Viremia was first detected in the infected group on d 7, peaked on d 14, and persisted at a high level until the end of the experiment. The control group tested negative for PCV3 throughout the study (Fig. 4A). PCV3 was detected in the heart, liver, spleen, lungs, and lymph nodes of the infected group, with the lymph nodes exhibiting the highest viral load (Fig. 4B). Histopathological examination showed that, compared to the control group, the experimental group exhibited more pronounced interstitial pneumonia in the lungs, with thickened alveolar septa; the lymph nodes exhibited a reduced number of lymphocytes and nuclear condensation; the liver showed extensive suppurative inflammatory cell infiltration in confluent areas; and there was an increase in the number of intestinal goblet cells. No significant pathological alterations were observed in the organs of the control group (Fig. 4C). Immunohistochemical (IHC) analysis showed the presence of brown PCV3-specific punctate deposits in the cytoplasm and nucleus of cells from the heart, liver, lungs, lymph nodes, spleen, and intestines, suggesting the presence of PCV3 antigen in these tissues (Fig. 4D). PBMCs were stimulated with ConA and VLPs containing the PCV3-Cap protein expressed in insect cells. The stimulation index (SI) was calculated by measuring the OD_450_ value. The results indicated that stimulation with ConA enhanced cell proliferation in both the infected and control groups, but there was no significant difference in the stimulation index between the two groups. However, after stimulation with VLP-PCV3-Cap, cell proliferation was inhibited, and the difference between the two groups was significant (*P* < 0.05), with a more pronounced inhibitory effect in the infected group (Fig. 4E).

### scRNA-seq

We used the 10× Genomics platform to perform 3′ scRNA-seq analysis of PBMCs from pigs in the PCV3-infected and control groups. A total of 53,297 cells were analyzed (PCV3-infected group: 25, 650; control cells: 27, 647), yielding 26 different fine clusters, which were visualized and displayed using t-distributed stochastic neighbor embedding (t-SNE) (Fig. S5A). Combined analysis using the Single Cell Cluster-based Annotation Toolkit for Cellular Heterogeneity (scCATCH) and Single-Cell Recognition (SingleR) was used to annotate 26 cell clusters, ultimately identifying 12 distinct cell types (Fig. S5B). Differentially highly expressed genes within the 12 categorized cell types were displayed using violin plots, which illustrate the distribution of key genes across different cellular subpopulations (Fig. S5C). This visualization provides a crucial reference and basis for further identification and study of these subpopulations.

**Fig 5:**
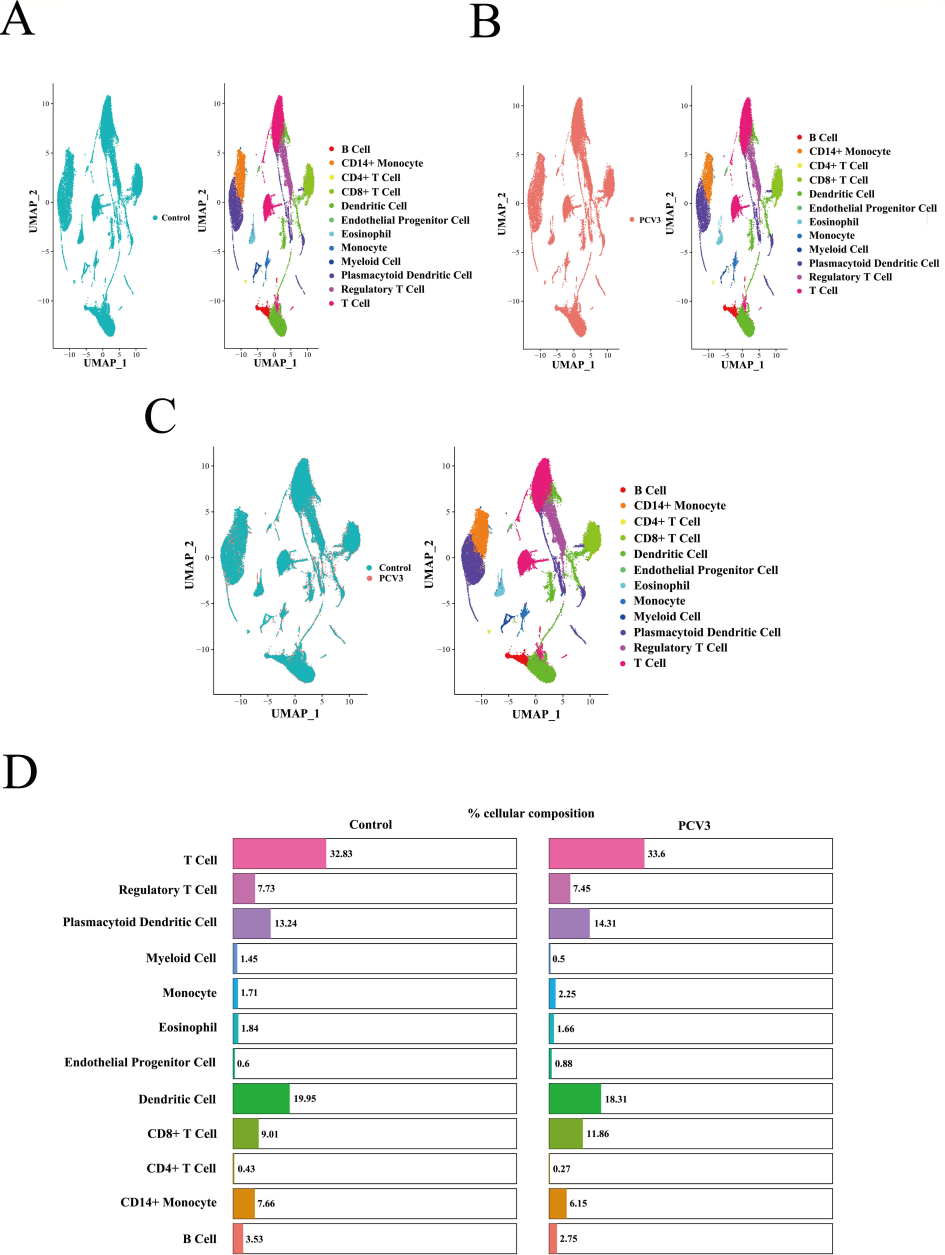
Single-cell profiling of cell populations in PBMCs. UMAP visualization of cell types in PBMCs from the control group (**A**) and the PCV3-infected group (**B**). (**C**) UMAP visualization comparing the heterogeneity of cell types between the control group and the PCV3-infected group. (**D**) The distribution of each cell type in the control group and the PCV3-infected group was represented as percentages.

Different cell types from the control and infected groups were visualized using uniform manifold approximation and projection (UMAP) (Fig. 5A–C), and the distribution of each cell type within the two groups is represented as a percentage (Fig. 5D). Analysis combining UMAP visualization and percentage distribution of cell types revealed no significant heterogeneity between the two groups; however, T cells had the highest proportion (32.83% for the control group and 33.6% for the PCV3 infection group), which may indicate that diversity, including various subgroups and functional characteristics, may exist. Subclustering of T cells facilitated the identification of specific T-cell subgroup changes within the infected group. Although the overall proportion of T cells was similar across groups, differences in subgroup ratios might indicate the impact of infection on the immune system. Therefore, to further investigate the differences between the two groups, we sub-populated the T cells by cell type.

### Reclassification and pseudotime analysis of T-cell subsets in PBMCs

**Fig 6:**
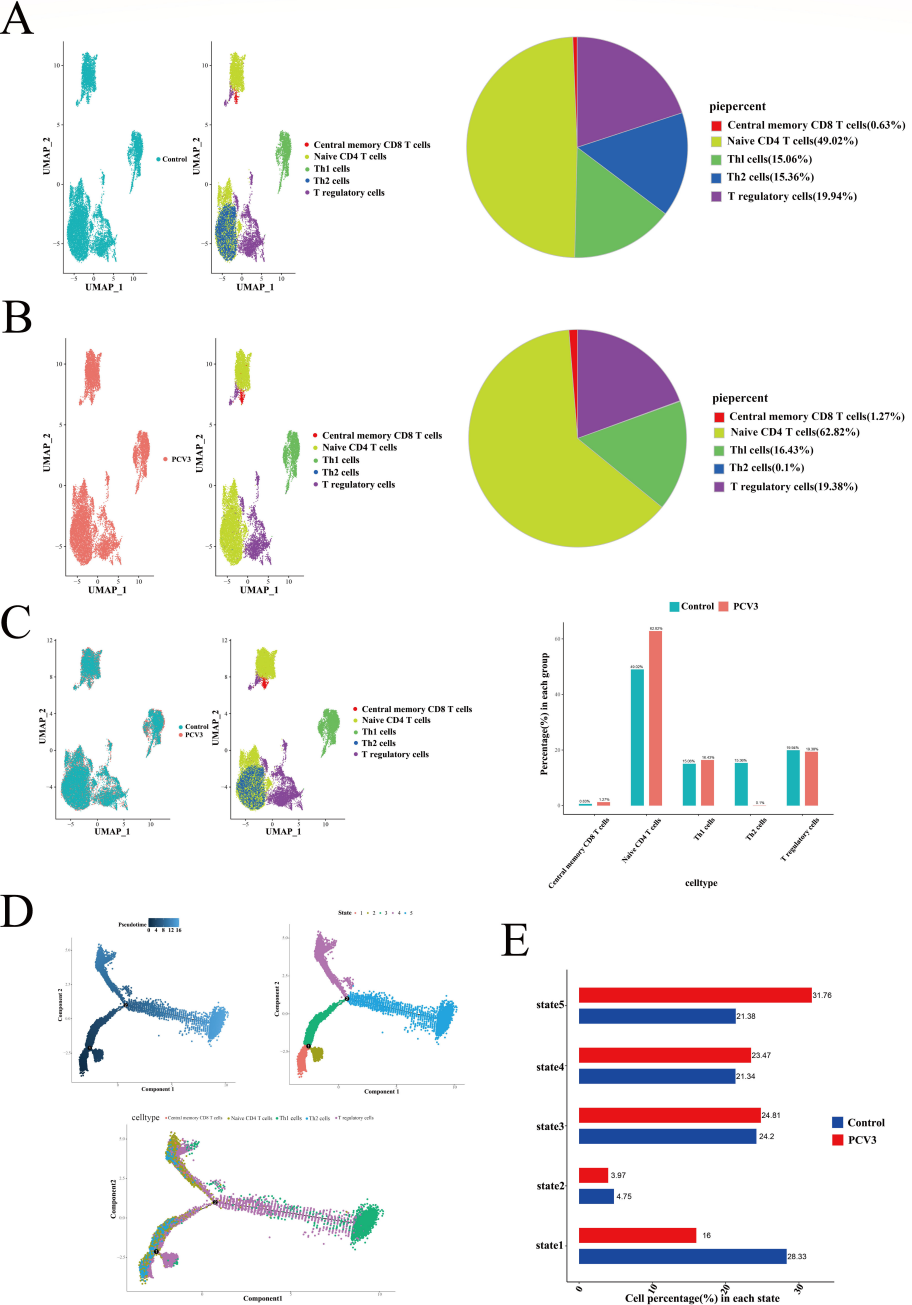
Reclassification and pseudotime analysis of T-cell subsets in PBMCs. UMAP and pie charts showing the cell subpopulations and proportions after T-cell repopulation in control (**A**) and PCV3-infected (**B**) PBMCs. (**C**) The heterogeneity of cell subpopulations between the control and infected groups was demonstrated with UMAP plots after re-clustering, and the number of cells in the different subpopulations is labeled with bar graphs. (**D**) Fitted-time analysis of the distribution of cell subpopulations across differentiation states. (**E**) Number of cells between samples in the five differentiation states.

After the reclassification of the T cells, visualization was performed using UMAP. Both the control and infected groups were divided into five T-cell subsets (Fig. 6A through C). In the control group, Th2 cells accounted for 15.36% of the population (Fig. 6A), while in the infected group, the proportion of Th2 cells was significantly reduced to 0.1% (Fig. 6B). However, the changes in central memory CD8+ T cells, native CD4+ T cells, Th1 cells, and T-regulated cells were not significant.

Using pseudotime analysis, T cells were classified into five differentiation states, with Th1 cells predominantly in the later stages of differentiation (Fig. 6D). This may be related to the virus’s ability to alter the host’s immune response, thereby suppressing the normal immune clearance process. In the control group, a greater number of cells were in State 1, whereas in the infected group, the majority of cells were in State 5 (Fig. 6E). Viral infection leads to the migration of immune cells, resulting in different differentiation states and distribution trajectories in the infected group compared to those in the control group.

## DISCUSSION

Oh et al. (39) collected pig inguinal lymph nodes solely infected with PCV3 for grinding and subsequent infection of primary kidney cells. However, in their study, viral propagation terminated after the eighth passage for unknown reasons. This isolation method fails to ensure viral purity and does not achieve a high titer of virus for use in pig infection. Mora-Díaz et al. (24) collected lungs, brains, and livers from mummified fetuses that tested positive for PCV3 but negative for other pathogens. The tissues were homogenized and inoculated into PK-15 cells. This method utilizes PK-15 cells for continuous passage but can only propagate up to the 9th passage with relatively low viral titers, making it unsuitable for large-scale production of PCV3 virus stock. In our study, PCV3 was able to be passaged continuously 25 times in PK-15 cells, overcoming the limitation of wPCV3, which could not be passaged continuously in cells. Nucleus fluorescence observation indicated that the transfected recombinant plasmid was transported into the cell nucleus and that rolling circle replication was successfully primed. PK-15 cells were infected with both PCV3 and wPCV3, and IFA revealed fluorescent signals in the cytoplasm, suggesting that PCV3 and wPCV3 can infect PK-15 cells but cannot enter the cell nucleus to complete their life cycles, which may explain the failure of virus isolation from these cells. Nocodazole treatment has shown that PCV3 replication is dependent on microtubule polymerization in the cell, similar to that of PCV2 (40).

Jiang et al. (41) used cDNA cloning technology to obtain the PCV3 virus, which was then used to infect specific pathogen-free (SPF) pigs at 4 and 8 weeks of age. They successfully replicated dermatitis and nephropathy syndrome. However, to date, no one has been able to reproduce these results. Temeeyasen et al. (21) infected CD/CD pigs with PCV3-positive tissues and did not observe significant clinical signs. In our study, after infecting CD pigs with PCV3, we observed persistent viremia. mRNA was extracted from the heart, liver, spleen, lungs, and lymph nodes, followed by reverse transcription to cDNA. Viral copy numbers were determined through RT-qPCR, and high viral loads were detected in these tissues. Histopathological examination revealed significant pathological changes in the lungs, lymph nodes, and liver. The lymph nodes exhibited a reduced number of lymphocytes, and nuclear condensation may be associated with the immunosuppression of PCV3. The above results showed that PCV3 can successfully infect CD pigs and cause characteristic pathological changes.

After stimulation of PBMCs with VLPs derived from the Cap protein expressed in insect cells, cell proliferation was significantly inhibited. This inhibition of PBMC proliferation may lead to an immune response delay, immune system functionality damage and dysregulation, increasing the susceptibility of pigs infected with PCV3 to other pathogens.

Following PCV3 infection, PBMCs were subjected to scRNA-seq on d 14 post-infection, and 12 distinct cell types were identified. Among them, T cells comprised the greatest proportion. Consequently, further sub-clustering of T cells revealed five cellular subtypes, with Th1 and Th2 cells representing distinct types of immune responses. Th1 cells primarily engage in cell-mediated immune responses, such as combating viral infections and tumors, whereas Th2 cells participate in humoral immunity and antibody production. A decrease in Th2 cell number while Th1 cells remain stable may suggest a shift in the immune response from Th2 to Th1. This shift, possibly induced by viral infection, could lead to an immune system imbalance. The virus may inhibit the generation of immune cells or induce early cells to enter the apoptosis stage, leading to a bias toward late-stage cell differentiation processes. Overall, in this study, the PCV3 virus was successfully obtained, and the first clear transmission electron microscope image was obtained.

## Data Availability

All data generated or analyzed during this study are included in this article and its supplemental material.

## SUPPLEMENTAL MATERIAL

Figure S1 jvi.00341-25-s0001.tif PCR detection of PK-15 cells harboring PCV3 at different passages.

Figure S2 jvi.00341-25-s0002.tif Viral copy number detection method.

Figure S3 jvi.00341-25-s0003.tif Infection of PK-15 cells with PCV3, as detected by IFA.

Figure S4 jvi.00341-25-s0004.tif Infection of PK-15 cells with wPCV3, as detected by IFA.

Figure S5 jvi.00341-25-s0005.tif Cell type analysis of cell clusters in PBMCs.

Supplemental legends jvi.00341-25-s0006.docx Legends for Fig. S1 to S5.

ASM does not own the copyrights to Supplemental Material that may be linked to, or accessed through, an article. The authors have granted ASM a non-exclusive, world-wide license to publish the Supplemental Material files. Please contact the corresponding author directly for reuse.

## References

[1] Todd D. 2000. Circoviruses: immunosuppressive threats to avian species: a review. Avian Pathol 29:373–394. doi:10.1080/030794500750047126 19184829

[2] Tan CY, Opaskornkul K, Thanawongnuwech R, Arshad SS, Hassan L, Ooi PT. 2020. First molecular detection and complete sequence analysis of porcine circovirus type 3 (PCV3) in Peninsular Malaysia. PLoS One 15:e0235832. doi:10.1371/journal.pone.0235832 32706778 PMC7380639

[3] Franzo G, Delwart E, Fux R, Hause B, Su S, Zhou J, Segalés J. 2020. Genotyping Porcine Circovirus 3 (PCV-3) nowadays: does it make sense? Viruses 12:265. doi:10.3390/v12030265 32121102 PMC7150946

[4] Palinski R, Piñeyro P, Shang P, Yuan F, Guo R, Fang Y, Byers E, Hause BM. 2017. A novel porcine circovirus distantly related to known circoviruses is associated with porcine dermatitis and nephropathy syndrome and reproductive failure. J Virol 91:e00143–17. doi:10.1128/JVI.01879-16 27795441 PMC5165205

[5] Li G, Wang H, Wang S, Xing G, Zhang C, Zhang W, Liu J, Zhang J, Su S, Zhou J. 2018. Insights into the genetic and host adaptability of emerging porcine circovirus 3. Virulence 9:1301–1313. doi:10.1080/21505594.2018.1492863 29973122 PMC6177243

[6] Kedkovid R, Woonwong Y, Arunorat J, Sirisereewan C, Sangpratum N, Lumyai M, Kesdangsakonwut S, Teankum K, Jittimanee S, Thanawongnuwech R. 2018. Porcine circovirus type 3 (PCV3) infection in grower pigs from a Thai farm suffering from porcine respiratory disease complex (PRDC). Vet Microbiol 215:71–76. doi:10.1016/j.vetmic.2018.01.004 29426409

[7] Kwon T, Yoo SJ, Park CK, Lyoo YS. 2017. Prevalence of novel porcine circovirus 3 in Korean pig populations. Vet Microbiol 207:178–180. doi:10.1016/j.vetmic.2017.06.013 28757021

[8] Assao VS, Santos MR, Pereira CER, Vannucci F, Silva-Júnior A. 2021. Porcine circovirus 3 in North and South America: epidemiology and genetic diversity. Transbound Emerg Dis 68:2949–2956. doi:10.1111/tbed.14238 34310859

[9] Xia D, Huang L, Xie Y, Zhang X, Wei Y, Liu D, Zhu H, Bian H, Feng L, Liu C. 2019. The prevalence and genetic diversity of porcine circovirus types 2. Arch Virol:2435–2449. doi:10.1007/s00705-019-04336-4 31273470

[10] Dhandapani G, Yoon SW, Noh JY, Jang SS, Han SH, Jeong DG, Kim HK. 2021. Detection of Porcine circovirus 3 from captured wild boars in Korea. Vet Med Sci 7:1807–1814. doi:10.1002/vms3.518 34057302 PMC8464250

[11] Prinz C, Stillfried M, Neubert LK, Denner J. 2019. Detection of PCV3 in German wild boars. Virol J 16:25. doi:10.1186/s12985-019-1133-9 30795772 PMC6387533

[12] Franzo G, Legnardi M, Hjulsager CK, Klaumann F, Larsen LE, Segales J, Drigo M. 2018. Full-genome sequencing of porcine circovirus 3 field strains from Denmark, Italy and Spain demonstrates a high within-Europe genetic heterogeneity. Transbound Emerg Dis 65:602–606. doi:10.1111/tbed.12836 29453822

[13] Sun W, Wang W, Xin J, Cao L, Zhuang X, Zhang C, Zhu Y, Zhang H, Qin Y, Du Q, Han Z, Lu H, Zheng M, Jin N. 2019. An epidemiological investigation of porcine circovirus 3 infection in dogs in the Guangxi Province from 2015 to 2017, China. Virus Res 270:197663. doi:10.1016/j.virusres.2019.197663 31301332 PMC7114628

[14] Wang T, Chai W, Wang Y, Liu W, Huang Z, Chen L, Guo R, Dong Y, Liu M, Zheng Q, Liu G, Wang C, Guo WP, Liu S, Li L. 2021. First detection and phylogenetic analysis of porcine circovirus 3 in female donkeys with reproductive disorders. BMC Vet Res 17:308. doi:10.1186/s12917-021-03013-6 34537035 PMC8449920

[15] Cui Y, Hou L, Pan Y, Feng X, Zhou jianwei, Wang D, Guo J, Liu C, Shi Y, Sun T, Yang X, Zhu N, Tong X, Wang Y, Liu J. 2022. Reconstruction of the evolutionary origin, phylodynamics, and phylogeography of the porcine circovirus type 3. Front Microbiol 13:898212. doi:10.3389/fmicb.2022.898212 35663871 PMC9158500

[16] Yang Z, Marthaler DG, Rovira A. 2022. Frequency of porcine circovirus 3 detection and histologic lesions in clinical samples from swine in the United States. J Vet Diagn Invest 34:602–611. doi:10.1177/10406387221099538 35674058 PMC9266519

[17] Ruiz A, Saporiti V, Huerta E, Balasch M, Segalés J, Sibila M. 2022. Exploratory study of the frequency of detection and tissue distribution of Porcine Circovirus 3 (PCV-3) in pig fetuses at different gestational ages. Pathogens 11:118. doi:10.3390/pathogens11020118 35215062 PMC8877316

[18] Kroeger M, Temeeyasen G, Piñeyro PE. 2022. Five years of porcine circovirus 3: what have we learned about the clinical disease, immune pathogenesis, and diagnosis. Virus Res 314:198764. doi:10.1016/j.virusres.2022.198764 35367483

[19] Dal Santo AC, Cezario KC, Bennemann PE, Machado SA, Martins M. 2020. Full-genome sequences of porcine circovirus 3 (PCV3) and high prevalence in mummified fetuses from commercial farms in Brazil. Microb Pathog 141:104027. doi:10.1016/j.micpath.2020.104027 32007620

[20] Vargas-Bermúdez DS, Vargas-Pinto MA, Mogollón JD, Jaime J. 2021. Field infection of a gilt and its litter demonstrates vertical transmission and effect on reproductive failure caused by porcine circovirus type 3 (PCV3). BMC Vet Res 17:150. doi:10.1186/s12917-021-02862-5 33832500 PMC8028087

[21] Temeeyasen G, Lierman S, Arruda BL, Main R, Vannucci F, Gimenez-Lirola LG, Piñeyro PE. 2021. Pathogenicity and immune response against porcine circovirus type 3 infection in caesarean-derived, colostrum-deprived pigs. J Gen Virol 102:10. doi:10.1099/jgv.0.001502 33206034

[22] Nguyen NH, Do DT, Nguyen TQ, Nguyen TT, Nguyen MN. 2021. Genetic diversity of porcine circovirus subtypes from aborted sow fetuses in Vietnam. Curr Microbiol 78:3751–3756. doi:10.1007/s00284-021-02641-3 34468854

[23] Sirisereewan C, Thanawongnuwech R, Kedkovid R. 2022. Current understanding of the pathogenesis of porcine Circovirus 3. Pathogens 11:64. doi:10.3390/pathogens11010064 35056012 PMC8778431

[24] Mora-Díaz J, Piñeyro P, Shen H, Schwartz K, Vannucci F, Li G, Arruda B, Giménez-Lirola L. 2020. Isolation of PCV3 from perinatal and reproductive cases of PCV3-associated disease and in vivo characterization of PCV3 replication in CD/CD growing pigs. Viruses 12:219. doi:10.3390/v12020219 32079070 PMC7077311

[25] Geng S, Luo H, Liu Y, Chen C, Xu W, Chen Y, Li X, Fang W. 2019. Prevalence of porcine circovirus type 3 in pigs in the southeastern Chinese province of Zhejiang. BMC Vet Res 15:244. doi:10.1186/s12917-019-1977-7 31307451 PMC6631677

[26] Tochetto C, de Lima DA, Varela APM, Ortiz LC, Loiko MR, Scheffer CM, Paim WP, Cibulski SP, Cerva C, Herpich J, Schmidt C, Franco AC, Mayer FQ, Roehe PM. 2020. Investigation on porcine circovirus type 3 in serum of farrowing sows with stillbirths. Microb Pathog 149:104316. doi:10.1016/j.micpath.2020.104316 32531497

[27] Deim Z, Dencső L, Erdélyi I, Valappil SK, Varga C, Pósa A, Makrai L, Rákhely G. 2019. Porcine circovirus type 3 detection in a Hungarian pig farm experiencing reproductive failures. Vet Rec 185:84. doi:10.1136/vr.104784 31177090

[28] Arruda B, Piñeyro P, Derscheid R, Hause B, Byers E, Dion K, Long D, Sievers C, Tangen J, Williams T, Schwartz K. 2019. PCV3-associated disease in the United States swine herd. Emerg Microbes Infect 8:684–698. doi:10.1080/22221751.2019.1613176 31096848 PMC6534263

[29] Krüger L, Längin M, Reichart B, Fiebig U, Kristiansen Y, Prinz C, Kessler B, Egerer S, Wolf E, Abicht J-M, Denner J. 2019. Transmission of porcine circovirus 3 (PCV3) by xenotransplantation of pig hearts into baboons. Viruses 11:650. doi:10.3390/v11070650 31315245 PMC6669873

[30] Wang S, Sun ST, Zhang XY, Ding HR, Yuan Y, He JJ, Wang MS, Yang B, Li YB. 2023. The evolution of single-cell RNA sequencing technology and application: progress and perspectives. IJMS 24:2943. doi:10.3390/ijms24032943 36769267 PMC9918030

[31] Herrera-Uribe J, Wiarda JE, Sivasankaran SK, Daharsh L, Liu H, Byrne KA, Smith TPL, Lunney JK, Loving CL, Tuggle CK. 2021. Reference transcriptomes of porcine peripheral immune cells created through bulk and single-cell RNA sequencing. Front Genet 12:689406. doi:10.3389/fgene.2021.689406 34249103 PMC8261551

[32] Zhang J, Liu J, Yuan Y, Huang F, Ma R, Luo B, Xi Z, Pan T, Liu B, Zhang Y, Zhang X, Luo Y, Wang J, Zhao M, Lu G, Deng K, Zhang H. 2020. Two waves of pro-inflammatory factors are released during the influenza A virus (IAV)-driven pulmonary immunopathogenesis. PLoS Pathog 16:e1008334. doi:10.1371/journal.ppat.1008334 32101596 PMC7062283

[33] Zhu L, Yang P, Zhao Y, Zhuang Z, Wang Z, Song R, Zhang J, Liu C, Gao Q, Xu Q, et al.. 2020. Single-cell sequencing of peripheral mononuclear cells reveals distinct immune response landscapes of COVID-19 and influenza patients. Immunity 53:685–696. doi:10.1016/j.immuni.2020.07.009 32783921 PMC7368915

[34] Fan B, Zhou J, Zhao Y, Zhu X, Zhu M, Peng Q, Li J, Chang X, Shi D, Yin J, Guo R, Li Y, He K, Fan H, Li B. 2023. Identification of cell types and transcriptome landscapes of porcine epidemic diarrhea virus-infected porcine small intestine using single-cell RNA sequencing. J Immunol 210:271–282. doi:10.4049/jimmunol.2101216 36548460

[35] Jiang B, Li L, Wu Y, Wang X, Gao N, Xu Z, Guo C, He S, Zhang G, Chen Y, Liu X, Li Z. 2024. Unveiling shared immune responses in porcine alveolar macrophages during ASFV and PRRSV infection using single-cell RNA-seq. Microorganisms 12:563. doi:10.3390/microorganisms12030563 38543614 PMC10974629

[36] Beach NM, Ramamoorthy S, Opriessnig T, Wu SQ, Meng XJ. 2010. Novel chimeric porcine circovirus (PCV) with the capsid gene of the emerging PCV2b subtype cloned in the genomic backbone of the non-pathogenic PCV1 is attenuated in vivo and induces protective and cross-protective immunity against PCV2b and PCV2a subtypes in pigs. Vaccine (Auckl) 29:221–232. doi:10.1016/j.vaccine.2010.10.050 21044670

[37] Cheung AK. 2015. Specific functions of the Rep and Rep׳ proteins of porcine circovirus during copy-release and rolling-circle DNA replication. Virology (Auckl) 481:43–50. doi:10.1016/j.virol.2015.01.004 25768890

[38] Zhan Y, Yu W, Cai X, Lei X, Lei H, Wang A, Sun Y, Wang N, Deng Z, Yang Y. 2021. Correction for Zhan et al., “The carboxyl terminus of the porcine circovirus type 2 capsid protein is critical to virus-like particle assembly, cell entry, and propagation”. J Virol 95:e00022–21. doi:10.1128/JVI.02409-20 PMC8104098 33846271

[39] Oh T, Chae C. 2020. First isolation and genetic characterization of porcine circovirus type 3 using primary porcine kidney cells. Vet Microbiol 241:108576. doi:10.1016/j.vetmic.2020.108576 31928694

[40] Cao J, Lin C, Wang H, Wang L, Zhou N, Jin Y, Liao M, Zhou J. 2015. Circovirus transport proceeds via direct interaction of the cytoplasmic dynein IC1 subunit with the viral capsid protein. J Virol 89:2777–2791. doi:10.1128/JVI.03117-14 25540360 PMC4325748

[41] Jiang H, Wang D, Wang J, Zhu S, She R, Ren X, Tian J, Quan R, Hou L, Li Z, Chu J, Guo Y, Xi Y, Song H, Yuan F, Wei L, Liu J. 2019. Induction of porcine dermatitis and nephropathy syndrome in piglets by infection with porcine circovirus type 3. J Virol 93:e00172–19. doi:10.1128/JVI.02045-18 30487279 PMC6363995

