# Supplementary figures and images for: Rescue of naïve porcine circovirus type 3 and its pathogenesis in CD pigs
Source: J Virol. 2025 May 12;99(6):e00341-25. doi: 10.1128/jvi.00341-25 (PMC12172495; doi:10.1128/jvi.00341-25)

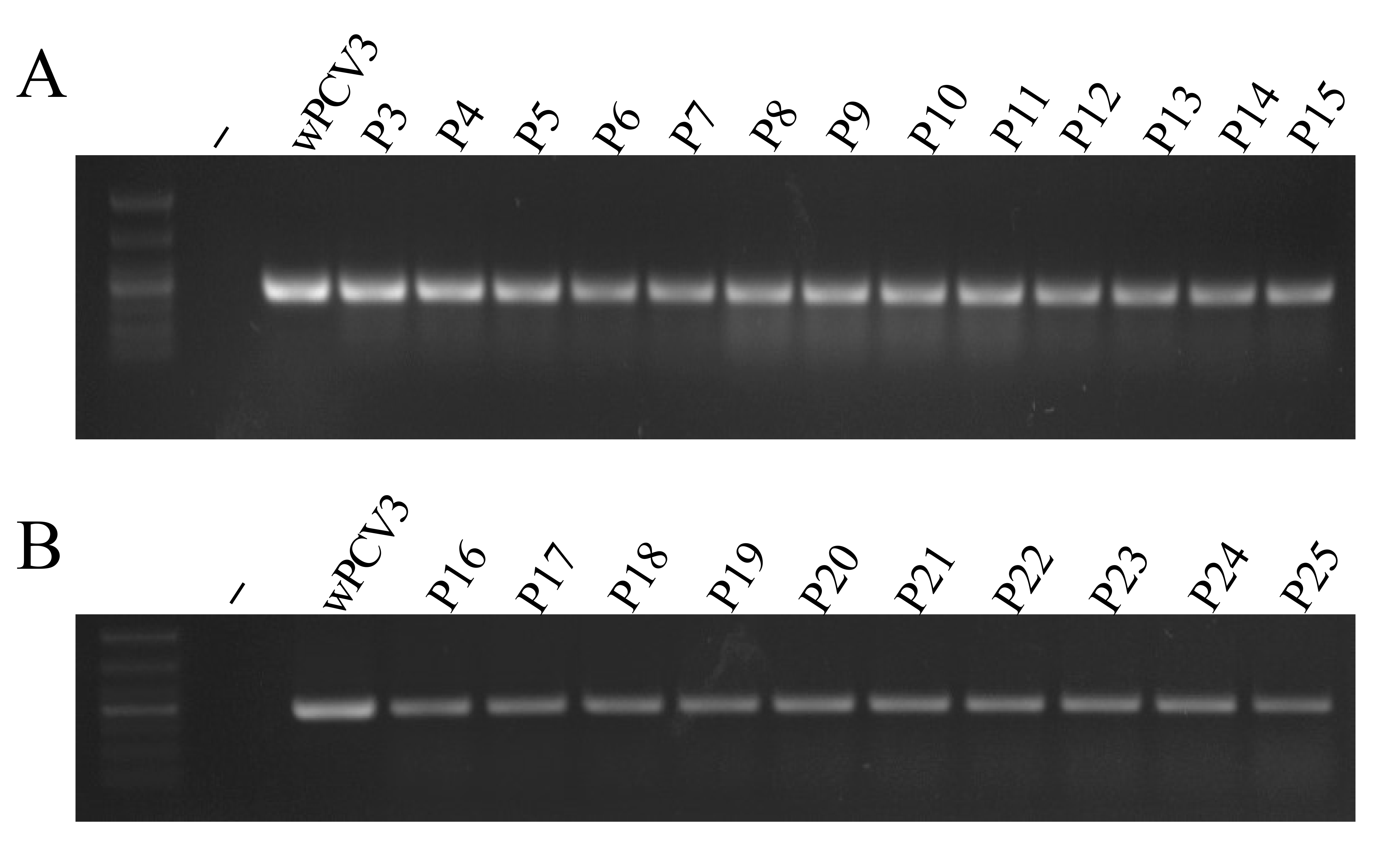

Supplement: Figure S1 — PCR detection of PK-15 cells harboring PCV3 at different passages. [file jvi.00341-25-s0001.tif]

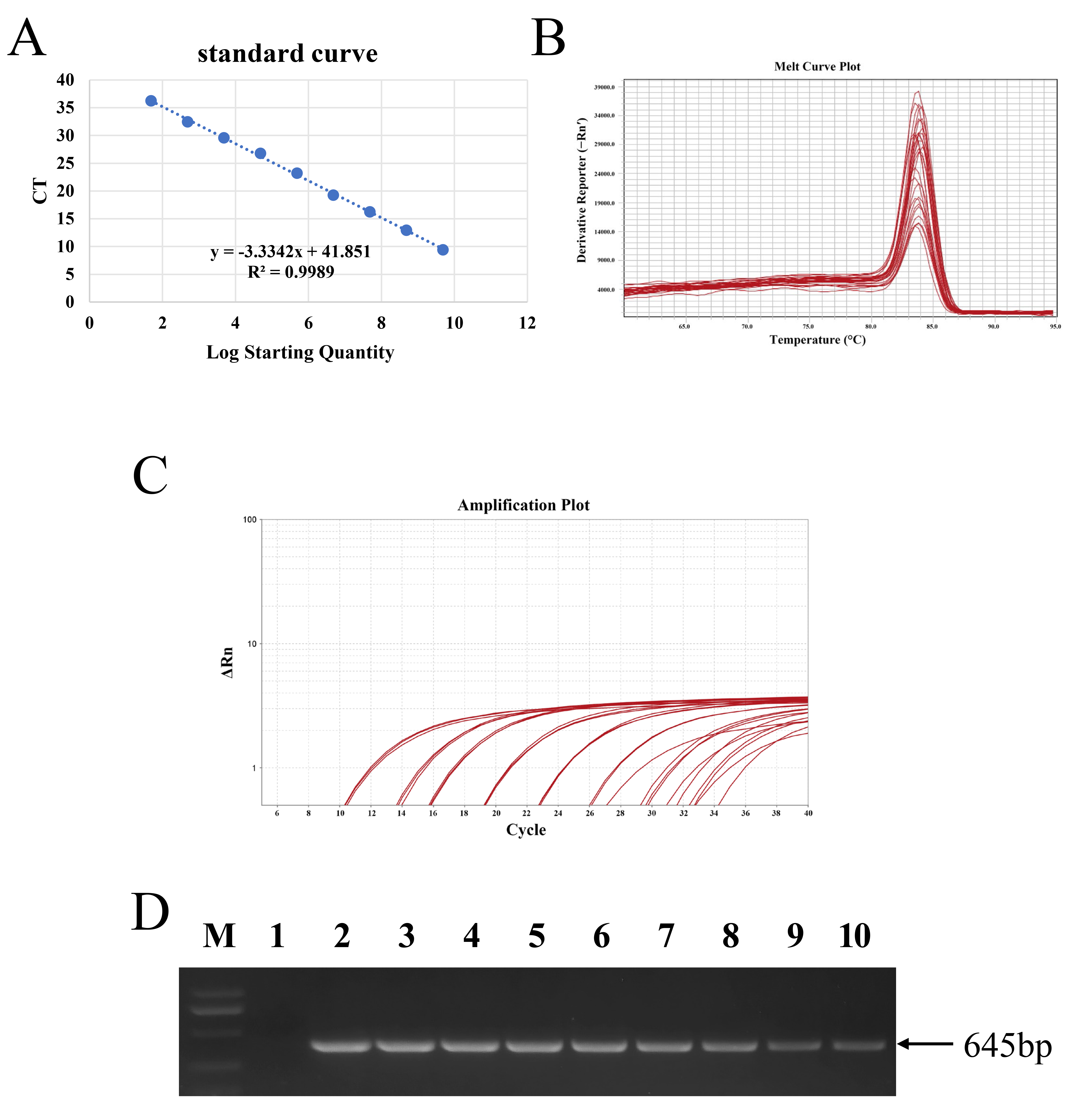

Supplement: Figure S2 — Viral copy number detection method. [file jvi.00341-25-s0002.tif]

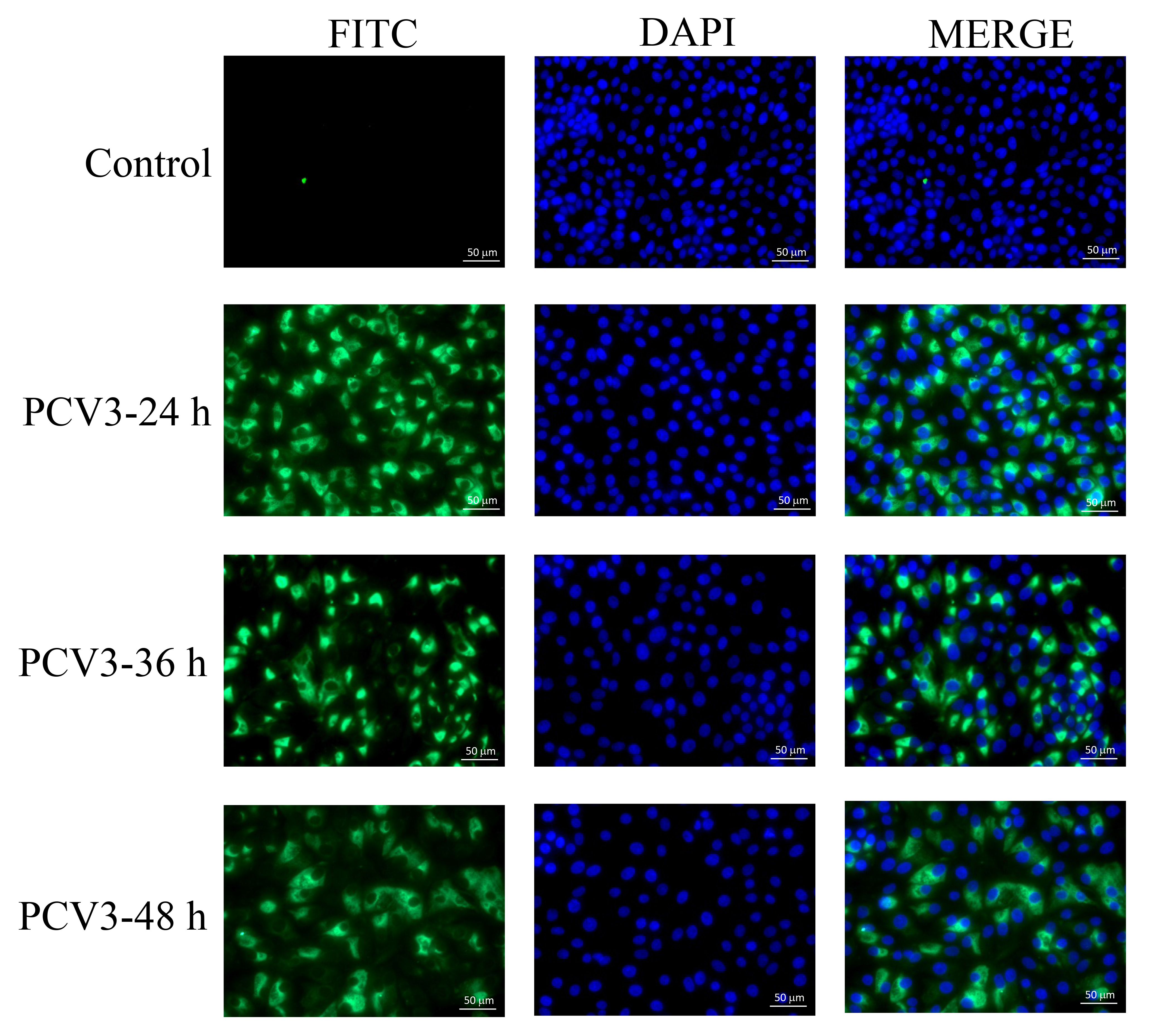

Supplement: Figure S3 — Infection of PK-15 cells with PCV3, as detected by IFA. [file jvi.00341-25-s0003.tif]

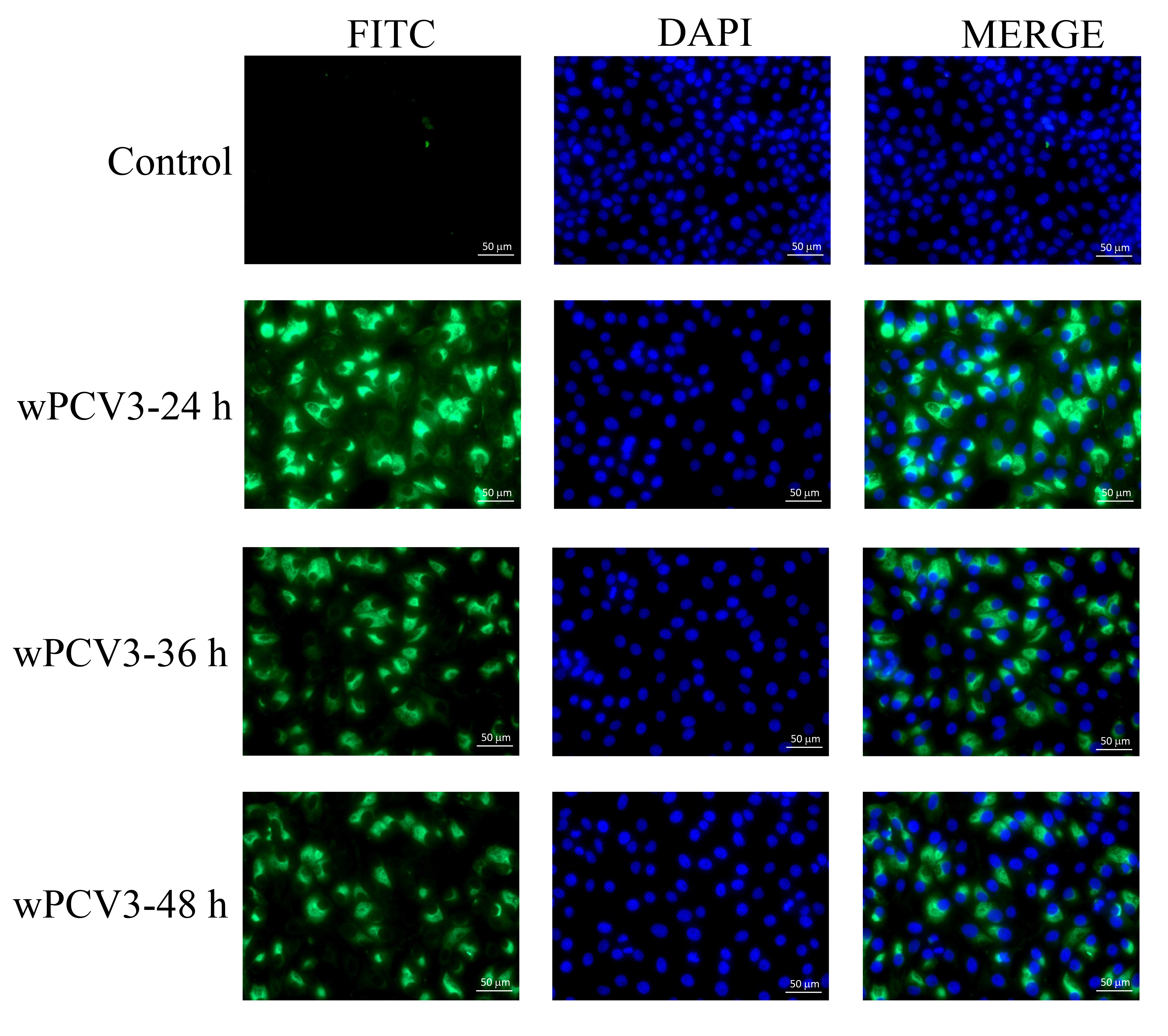

Supplement: Figure S4 — Infection of PK-15 cells with wPCV3, as detected by IFA. [file jvi.00341-25-s0004.tif]

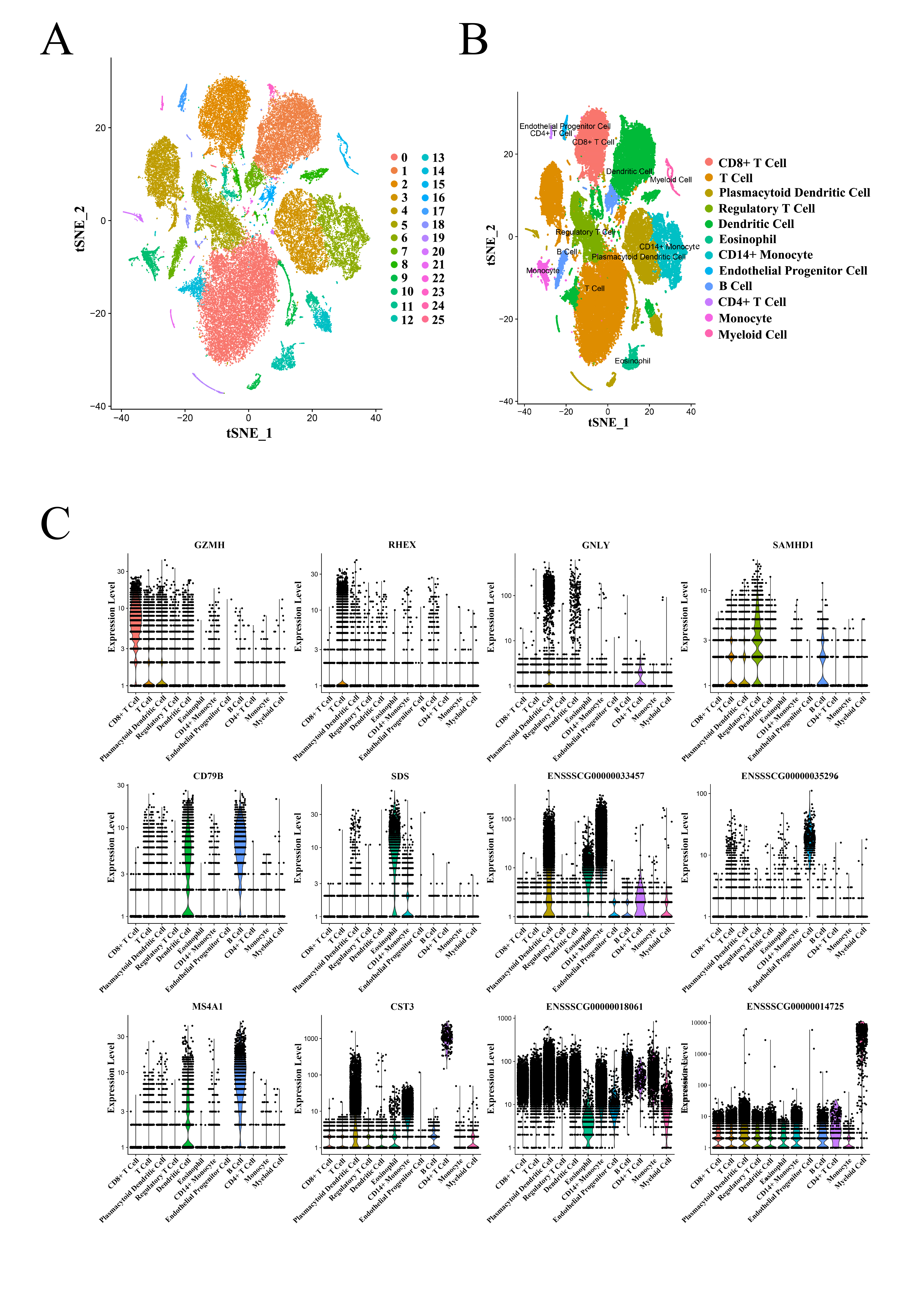

Supplement: Figure S5 — Cell type analysis of cell clusters in PBMCs. [file jvi.00341-25-s0005.tif]
