# Supplementary material for: Rescue of naïve porcine circovirus type 3 and its pathogenesis in CD pigs
Source: J Virol. 2025 May 12;99(6):e00341-25. doi: 10.1128/jvi.00341-25 (PMC12172495; doi:10.1128/jvi.00341-25)
Supplement: Supplemental legends — Legends for Fig. S1 to S5. [file jvi.00341-25-s0006.docx]

**Supplementary Figure 1.** PCR detection of PK-15 cells harboring PCV3 at different passages. (A and B) PK-15 cells transfected with recombinant plasmids were continuously passaged, and even at the 25th passage, specific bands could still be detected by PCR.

**Supplementary Figure 2.** Viral copy number detection method. (A and B) The linear equation of the standard curve was determined to be y = -3.3342x + 41.851, with a correlation coefficient (R²) of 0.9989. (C) The lowest detectable copy number by real-time fluorescence quantitative PCR was 4.8 copies/μl. (D) 1. Control; 2-10. Standard plasmids with copy numbers ranging from 4.8×10^10^ to 4.8×10^2^ copies were used. The lowest detectable copy number of the standard plasmid by conventional PCR was 4.8×10^2^ copies/μl.

**Supplementary Figure 3.** Infection of PK-15 cells with PCV3, as detected by IFA. Cytoplasmic fluorescence (green) was observed in PCV3-infected PK-15 cells at 24 h, 36 h, and 48 h post infection using a 2E6 monoclonal antibody, while the cell nuclei were stained with DAPI (blue).

**Supplementary Figure 4.** Infection of PK-15 cells with wPCV3, as detected by IFA. Cytoplasmic fluorescence (green) was observed in wPCV3-infected PK-15 cells at 24 h, 36 h, and 48 h post infection using a 2E6 monoclonal antibody, while the cell nuclei were stained with DAPI (blue).

**Supplementary Figure 5.** Cell type analysis of cell clusters in PBMCs. Visualization of 26 clusters (A) and 12 cell types (B) in PBMCs using a t-SNE plot. (C) Visual representation of differentially expressed genes with high expression among the 12 predefined cell types using violin plots, with annotation of the distribution of key genes across different cell subgroups.
